# Supplementary material for: Problems with using comparative analyses of avian brain size to test hypotheses of cognitive evolution
Source: PLoS One. 2022 Jul 22;17(7):e0270771. doi: 10.1371/journal.pone.0270771 (PMC9307164; doi:10.1371/journal.pone.0270771)
Supplement: Attachment — Submitted filename: Problems with comparative anlyses of avian brain size - recommendation.docx [file pone.0270771.s007.docx]

Original Submission:

1.1 Recommendation

Revise Major

2. Comments to Author

Ms. Ref. No.: PONE-D-22-00405

Title: Problems with comparative analyses of avian brain size

Overview and general recommendation:

Brilliant job! A very intriguing and eye-opening manuscript to the issues around the different analyses. I thoroughly enjoyed reading and reviewing it. The methodology was beautifully written and very detailed to allow a straightforward replication if required by others. The methodology appeared to suit the aims which was justified in the discussion section. The methodology had a diverse range of variables used to justify the aims which displays a lot of thought went into this study/manuscript. However, there were some sections which were a bit difficult to read. For example, the aims were jumbled around in the introduction rather than in a separate paragraph, so it was a bit tricky to tease out the aims. It would be useful if this was addressed. Otherwise, it was a great manuscript with some logical suggestions to improve this area of research.

2.1 Major comments:

- It was a bit difficult to tease apart the aims. One aim was mentioned in line 61 about “Here we interrogate the potential pitfalls…”. I got the idea that you were about to go into the method section immediately after that sentence as you just stated an aim. However, there was another paragraph about brain size and cognitive ability afterwards as it was still part of the introduction. There was another similar situation in line 73 with a sentence starting with “Here, we collate data from multiple datasets…”. I understand where you are coming from and why you wanted to place some aims there as it matches the paragraph topic, but maybe there should be a separate paragraph at the end of the introduction and before the methods which is solely on the aims. That way you can clearly define your aims for the reader. This is just an example of how you could write it:

“*Aims:*

Aim 1 – to interrogate the potential pitfalls of…

Aim 2 – to interrogate the brain estimates across datasets…”

This will help a lot with the flow and create an easier read for the readers.

I also notice you mention the aims again in lines 102 and 103 so maybe no need to mention it in line 73 and 61? Please readjust the aim section to make it more understandable.

- Line 93 to line 101 – this sounds very much like a methodology section, but it is located in the introduction. I suggest shifting this section to the methodology section and entitle it “study species” or something similar.
- The introduction into the topic of cognition and brain sizes was good. Maybe some more examples/references of the SIH and EIH in different animal groups could be used before primates are mentioned in line 51. This could give the readers a better understanding of the research around this topic and reinforce the idea that the two different cognitive comparative studies can produce different outcomes.
- Title – the title is great and suits the manuscript, but maybe you could add something about cognitive ability into it. This is since you reference it multiple times in the manuscript and if I am correct, are trying to see how robust the different methodologies are by testing for it using the brain size as a proxy.
- Line 252, 254 and 256 – just the species name was used such as *Pyrrhura frontalis* and *Acanthorhynchus tenuirostris.* Please use the common names as well since some readers may not know what animal you are referring to. You can add the species name beside the common name immediately after. For example, you could write line 252 with “…the minimum and maximum brain size estimates of the maroon-bellied parakeet, *Pyrrhura frontalis,* overlapped…” or use a similar method you used in line 201 and 202. It makes it a bit easier for readers who do not know what the common name of the *Pyrrhura frontalis* is so they know which bird you are talking about. This is considering I too had no idea what that bird or the others mentioned in line 254 and 256 were until I googled it.

2.2 Minor comments:

- Line 37 – in the abstract section, can you please specify/modify the phrase “direct measures” to explain what it is.
- Line 289 and 290 – these are good tables but, if possible, please fit one table each onto a single page? Maybe making the landscape orientation for the page specifically for the table horizontal? Then it can go back to being vertical for the writing section.
- Line 290 – in table 2, column λ, please make the numbers horizontal.
- Some of the paragraphs in the discussion section are very long. In line 322-343, please separate it into separate paragraphs. Also, the paragraph in lines 344-376 is extremely long. Please separate it into different chunks for a smoother read.
- Line 97 – the “Corvides infraorder” was mentioned. Please explain what a Corvide infraorder is so readers who do not know will know that it is.
