# Supplementary material for: Problems with using comparative analyses of avian brain size to test hypotheses of cognitive evolution
Source: PLoS One. 2022 Jul 22;17(7):e0270771. doi: 10.1371/journal.pone.0270771 (PMC9307164; doi:10.1371/journal.pone.0270771)
Supplement: Attachment — Submitted filename: Problems with using comparative anlyses of avian brain size to test hypotheses of cognitive evolution - recommendation.docx [file pone.0270771.s010.docx]

Research Article:

1.1 Recommendation

Revise Minor

2. Comments to Author

Ms. Ref. No.: PONE-D-22-00405R1

Title: Problems with using comparative analyses of avian brain size to test hypotheses of cognitive evolution

Overview and general recommendation:

This is coming along nicely! I enjoyed re-reading it and reviewing it again as it has improved so much and reads a lot better. I love the new additions to it and it is much more clear and easier to read. I just have a few comments and suggestions mainly regarding some grammar but other than that I think it is a great paper which can improve the understanding of avian brain size and cognitive evolution.

2.1 Minor comments:
- Please insert a ‘:’ after the italic subheading and before the first word of the sentence in lines 103, 118, 136, 138, 145, 154, 166, 169, 175, 194, 211, 218, 224, 238, 255 and 293.

For example, you could insert the “:” like this in line 136 “***Variables:*** We extracted/collated”.

- Line 105 – Add “s” to bird so it is plural i.e., “species of birds”.
- Line 167 – you end a sentence mentioning “for seven diet types”. Could you please elaborate on those seven diet types and the range of values i.e., is it 1-10 or 1-50.
- Line 170-171 – can you define in briefly “social foraging’ and “cooperative breeding”
- Line 321 – Table 2 – I am assuming that the significant results are in bold. Could you please indicate in the table title if that is correct.
- Line 367 – change “madd” to “mass”
