# Supplementary material for: Problems with using comparative analyses of avian brain size to test hypotheses of cognitive evolution
Source: PLoS One. 2022 Jul 22;17(7):e0270771. doi: 10.1371/journal.pone.0270771 (PMC9307164; doi:10.1371/journal.pone.0270771)
Supplement: Attachment — Submitted filename: Response to reviewers_Rev2.docx [file pone.0270771.s011.docx]

**Response to Reviewers**

**Reviewer 1**

Overview and general recommendation:

This is coming along nicely! I enjoyed re-reading it and reviewing it again as it has improved so much and reads a lot better. I love the new additions to it and it is much more clear and easier to read. I just have a few comments and suggestions mainly regarding some grammar but other than that I think it is a great paper which can improve the understanding of avian brain size and cognitive evolution.

*We are so glad you enjoyed re-reading it and feel that it has improved following revision. Thank you for your time in helping us to improve this manuscript. Below we have addressed your specific points in this review round:*

2.1 Minor comments:
- Please insert a ‘:’ after the italic subheading and before the first word of the sentence in lines 103, 118, 136, 138, 145, 154, 166, 169, 175, 194, 211, 218, 224, 238, 255 and 293.

For example, you could insert the “:” like this in line 136 “***Variables:*** We extracted/collated”.

*Good suggestion – we have done this.*

- Line 105 – Add “s” to bird so it is plural i.e., “species of birds”.

  *We rephrased to “bird species”.*
- Line 167 – you end a sentence mentioning “for seven diet types”. Could you please elaborate on those seven diet types and the range of values i.e., is it 1-10 or 1-50.

  *We have now added an explanation of what the seven diet types are, and the range of values in our sample.*
- Line 170-171 – can you define in briefly “social foraging’ and “cooperative breeding”

*We have now added brief definitions of these terms.*

- Line 321 – Table 2 – I am assuming that the significant results are in bold. Could you please indicate in the table title if that is correct.

  *Yes – we added this to the caption.*
- Line 367 – change “madd” to “mass”

*Thank you for catching this – it is now corrected.*

**Reviewer 2**

Again, It was a pleasure to read your work. I ‘m glad you have considered all my suggestions and found them helpful in improving your work. I think that you have made a great effort to address all comments and suggestions from the other reviewers and me, and I consider your work will add to the debate on the cognitive evolution in birds. After reading the MS, I found no more issues but recommend you review it again for any minor details.

Congratulations on your great job, first developing such an exciting idea, then improving your MS.

*Thank you, we are glad that you enjoyed reading our manuscript and thank you for your helpful and insightful review!*

**Reviewer 3**

I thank the authors for their detailed response and for addressing all comments made. I can see an improvement in the text and especially in the discussion. I also think the feedback from the fourth reviewer helped improve it as well, and I'm glad you were able to catch some of the issues with the dataset in time.

*Thank you for your detailed and insightful review. We agree that R4’s feedback was also very useful, and are glad that you see an improvement in our manuscript following revisions.*
